# Audiometric Hearing and Plasma Biomarkers of Alzheimer’s Disease Pathology and Neurodegeneration in the ARIC-NCS

**DOI:** 10.1093/geroni/igaf122.1080

**Published:** 2025-12-31

**Authors:** Jennifer Deal, James Pike, Priya Palta, Nicholas Reed

**Affiliations:** Johns Hopkins Bloomberg School of Public Health, Baltimore, Maryland, United States; New York University, New York, New York, United States; UNC School of Medicine, Chapel Hill, North Carolina, United States; New York University Grossman School of Medicine, New York, New York, United States

## Abstract

The mechanism linking hearing loss to Alzheimer’s disease and related dementias (ADRD) is unknown. We investigated cross-sectional associations of hearing loss with plasma biomarkers of AD and neurodegeneration, hypothesizing that hearing loss is associated with biomarkers reflective of broader neurodegeneration but not with AD-specific markers. Data is from the Atherosclerosis Risk in Communities Neurocognitive Study. Pure tone air conduction hearing thresholds (frequencies 0.5-4 kHz) were obtained at Visit 6 (2016-17) and averaged, with better-ear hearing loss modeled continuously. The Quanterix SiMoA platform measured plasma biomarkers from stored specimens at the nearest visit – Visit 5 (2011-13), Visit 6 (2016-17) or Visit 7 (2018-19) – including amyloid-β 42 /amyloid-β 40 ratio, phosphorylated tau (p-tau) 181, neurofilament light chain (NfL), and glial fibrillary acidic protein (GFAP). Multivariable-adjusted linear regression estimated differences in biomarker levels, adjusting for time between audiology assessment and plasma collection, age, sex, race, center, education, APOE e4 genotype, BMI, estimated glomerular filtration rate, cigarette use, total cholesterol, high-density lipoprotein cholesterol, hypertension, diabetes, coronary heart disease and hearing aid use. Participant were 67-94 years, 60% female and 36% self-identified identified Black race. Hearing loss was positively associated with NfL, with each 10 dB increase (worse hearing) associated with 0.079 (95% confidence interval [CI] 0.007, 0.150) higher log NfL. Poorer audiometric hearing in older adults is associated with plasma biomarkers of neurodegeneration, specifically NfL, but is not associated with AD-specific markers (amyloid and tau), suggesting that pathways linking hearing and ADRD are independent of these pathognomonic Alzheimer’s-related brain changes.

